# Ethnic background and differences in health care use: a national cross-sectional study of native Dutch and immigrant elderly in the Netherlands

**DOI:** 10.1186/1475-9276-8-35

**Published:** 2009-10-08

**Authors:** Semiha Denktaş, Gerrit Koopmans, Erwin Birnie, Marleen Foets, Gouke Bonsel

**Affiliations:** 1 Department of Health Policy and Management, Erasmus Medical Centre, PO Box 1738, 3000 DR Rotterdam, The Netherlands

## Abstract

**Background:**

Immigrant elderly are a rapidly growing group in Dutch society; little is known about their health care use. This study assesses whether ethnic disparities in health care use exist and how they can be explained. Applying an established health care access model as explanatory factors, we tested health and socio-economic status, and in view of our research population we added an acculturation variable, elaborated into several sub-domains.

**Methods:**

Cross-sectional study using data from the "Social Position, Health and Well-being of Elderly Immigrants" survey, conducted in 2003 in the Netherlands. The study population consisted of first generation immigrants aged 55 years and older from the four major immigrant populations in the Netherlands and a native Dutch reference group. The average response rate to the survey was 46% (1503/3284; country of origin: Turkey n = 307, Morocco n = 284, Surinam n = 308, the Netherlands Antilles n = 300, the Netherlands n = 304).

**Results:**

High ethnic disparities exist in health and health care utilisation. Immigrant elderly show a higher use of GP services and lower use of physical therapy and home care. Both self-reported health status (need factor) and language competence (part of acculturation) have high explanatory power for all types of health services utilisation; the additional impact of socio-economic status and education is low.

**Conclusion:**

For all health services, health disparities among all four major immigrant groups in the Netherlands translate into utilisation disparities, aggravated by lack of language competence. The resulting pattern of systematic lower health services utilisation of elderly immigrants is a challenge for health care providers and policy makers.

## Background

Europe's history is one of emigration and immigration. Half a century ago West European countries witnessed the arrival of the first labour immigrants and immigrants from (former) colonies. By now, these groups have come to age and as remigration is a rare event, the number of aged immigrants (age 55 years and older) is rapidly rising. In the Netherlands, the proportion of older immigrants will grow from 7.2% in 2003 to 14.6% in 2020 in the immigrant population [1]. The largest groups came from Turkey, Morocco, Surinam, and the Dutch Antilles in the 60ties and 70ties of the 20th century. Turkish and Moroccan people moved to the Netherlands as labour migrants. Surinamese and Antilleans came to the Netherlands primarily for higher education and as a result of decolonisation.

In developed countries, health care utilisation between immigrant and indigenous groups differs [2-10]. Lower use of specialised health care has been observed, in particular if actual need and social position are taken into consideration [7]. Studies in the Netherlands show a similar pattern of decreased utilisation of clinical care [4]. However, some immigrant groups visit their GP more frequently than the native Dutch [e.g. [4,11]]. Available studies are often limited to selected immigrant groups, to populations in large cities, and focus on one type of health care service. Moreover, the explanatory role of cultural and socio-economic factors is not or only partially elaborated on [12-14] and differences in health are usually not separated from differences in health care utilisation. Consequently the 'ethnic factor' in health care utilisation remains an enigma, and this black box position hampers evidence based improvement of both inequities in health and health care use, to the extent that these are present. The Andersen model, an established model of access to health care, offers tools to study health services utilisation [15].

In this study, we will investigate to what extent utilisation differences between elderly among the four largest immigrant groups in the Netherlands and native elderly can be explained by health status and by socio-economic factors, and whether remaining differences can further be explained by acculturation and ethnic background.

## Methods

### Conceptual model

**Figure 1:**
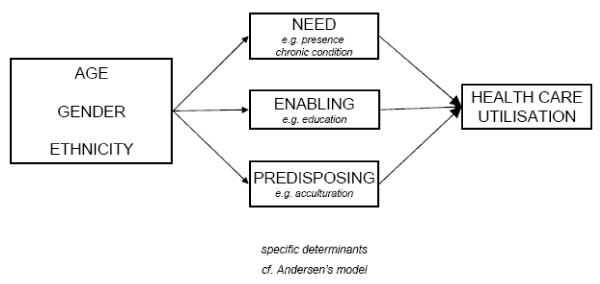
**Adapted Andersen model**.

We used Andersen's behavioural model as a framework to study health services utilisation (see Figure 1).

The model structure rests on three individual determinants of health care use, which we elaborate below, illustrated by Dutch immigrant examples.

(1) Need, which refers to ill-health conditions or deficits in health status. Especially self-perceived health is relevant here, since it initiates the decision to seek care. Most elderly immigrants perceive their health worse then natives and they experience more problems in Activities in Daily Living (ADL), pain, chronic conditions and a worse mental health [4,16,17]. (2) So-called enabling factors, which reflect the economic means (e.g., income) and human capital (e.g., education, knowledge) which enable people to use health services. In this context a lower socioeconomic position implies less knowledge on available services, less financial resources, and less self-reliance. In the Netherlands, first generation elderly immigrants from Turkey and Morocco are low educated and women often are illiterate [16]. Turkish and Moroccan elderly often have been unemployed for a long time and consequently have low income. Compared to Turkish and Moroccan elderly, Surinamese and Antillean elderly are better off resulting in an intermediate social economic position [18]. (3) Predisposing factors, the third determinant group, refers to the propensity of individuals to use services, including beliefs and attitudes regarding health and use of specific services. In the context of migrant use of health services, these attitudes primarily are a function of acculturation [19]. The general concept of acculturation, including acculturation in the domain of health care, is defined as "those phenomena which result when groups of individuals having different cultures come into continuous first-hand contact, with subsequent changes in the original cultural pattern of either or both groups" [20]. As our paper is focussed on migrant use of health services, we added to the Andersen model two complementary operationalisations of acculturation, derived from Berry and Ester respectively. Berry [21] articulates the process of any migrant's acculturation into two decisions. The first pertains to the decision whether one maintains his or her own cultural identity. The second one involves the decision whether to engage in relations (contact and participation) within the larger society. Both decisions can co-exist, and strongly relate to (acquired) language proficiency. The gradual adaptation to modernity can be considered a part of acculturation. 'Modernity' in Ester's [22] view is the most fundamental feature of Western societies and is defined as the transition of an agricultural to an (post)industrial society characterized by individualisation, secularisation, pluralisation, emancipation and democratisation [23,24]. Most of these processes also apply to health care. The dominant migrant groups in the Netherlands show different patterns of modernisation according to their background and generation [25,26].

### Data source and population

We used data from the "Social Position, Health and Well-being of Elderly Immigrants" survey, conducted in 2003 in the Netherlands [16,27]. To achieve a truly representative sample, first, on the basis of municipality and region size, all municipalities in the Netherlands were classified into 16 strata with different percentages of immigrant persons. From these 16 strata, 9 strata with the highest percentage of the immigrants were selected. Secondly, within the 9 strata, for each migrant group separately, the 11 municipalities with the largest prevalence of that particular migrant group were selected; ex post this strategy emerged into the same 11 municipalities, with, of course, slightly different patterns of ethnicity prevalences. This method has been used in large household surveys among immigrants in the Netherlands [11]. Samples were drawn from the municipal population registers. Ethnic background was established by country of birth, as documented in these registers. Compared to the Dutch population, immigrant elderly are less represented in the oldest age groups, while men are overrepresented because e.g. not all male immigrants were reunited with their spouses in the host country. Therefore, the sample was stratified into sex and two age groups (55-64 years and 65 years and older) and equal numbers per stratum were randomly selected. A total sample of 3284 people (808 Turks; 455 Moroccans; 688 Surinamese, 636 Antilleans and 697 Dutch) aged 55 years and above was drawn from the municipal registers. Of the 3284 subjects sampled, 1503 completed the questionnaire. The response rates were amongst Turkish 43.6%, Moroccans 65.3%, Surinamese 48.7%, Antilleans 54.2% and amongst native Dutch 47.3%. Excluding those with incorrect home addresses (amongst Turkish 5.6%, Moroccans 2.9%, Surinamese 3.9%, Antilleans 7.1% and Dutch 3.7%), the reasons for non-response were the following: (1) respondents could not be reached during the fieldwork: amongst Turkish 35.0%, Moroccans 16.2%, Surinamese 21.1%, Antilleans 22.7% and amongst Dutch 10.9%; (2) language problems: amongst Turkish 3.5%, Moroccans 0.7%, Surinamese 0.4%, amongst Antillean and Dutch 0%; (3) some elderly considered themselves too ill: amongst Turkish 6.7%, Moroccans 3.5%, Surinamese 8.4%, Antilleans 6.9% and amongst Dutch 8.6%; (4) respondents refused participation: amongst Turkish 11.3%, Moroccans 13.8%, Surinamese 21.4%, Antilleans 16.2% and amongst Dutch 33.1%; and finally other specified reasons: amongst Turkish 0.5%, Moroccans 0.4% and amongst Surinamese, Antilleans and Dutch 0%.

### Data collection method

The survey was translated into Turkish and Moroccan Arab and extensively tested in a pilot study. For the primary study 202 interviewers were trained: 61 native Dutch, 19 Antillean, 50 Moroccan, 27 Surinamese and 45 Turkish. Between April 2003 and December 2003, data collection took place: trained interviewers from a similar ethnic background conducted structured face-to-face interviews at home. The respondents were approached personally on their home addresses for two reasons: (1) to enhance participation and explain any respondent's questions raised on the aims and procedures of the study, and (2) possession and/or the proportion of secret telephone numbers among some ethnic groups are at a low respectively high level. For the approach of respondents interviewers were instructed to pay visits during daytime and evening to avoid work-related non-response. If the respondent was absent, the interviewer was instructed to re-visit the same address at least two times. All respondents received a 5,- euro's gift certificate. Reluctance to participate was related to not being convinced of the usefulness, apparent oversampling of immigrant groups for other studies, and a changing societal context which was clearly less tolerant towards immigrants.

### Measurements

Utilisation of five types of health care (yes/no) was investigated: (1) GP and (2) specialist consultations [frequency in the past two months], (3) physical therapy [at least one session of in the past 12 months], (4) hospital admission [at least one overnight stay in the past 12 months], and (5) home care [any use in the past 5 years].

Three indicators of health status (need factors) were included: self-rated health measured by the single-item question 'In general would you describe your health as: excellent, very good, good, poor, very poor', subsequently dichotomised into very poor/poor and good/very good/excellent [28]; the number of self-reported chronic conditions (ranging from 0 to 11) from which the respondents suffered in the 12 months preceding the interview [checklist of conditions is part of the Dutch national health survey [29]]; mental health as measured by the SF-12 Mental Component Summary (MCS). Mental health was covered by four questions referring to the past 4 weeks: (1) Have you felt calm and peaceful? (All of the time, most of the time, a good bit of the time, some of the time, a little of the time, or none of the time); (2) Did you have a lot of energy?; (3) Have you felt downhearted and blue?; (4) How much of the time has your physical health or emotional problems interfered with your social activities like visiting with friends, relatives etc.? Sum scores have a range of 0 to 100 [28,30].

Indicators of socio-economic position (enabling factors) were educational level and household income [31]. Educational level concerned the highest degree achieved (no education/primary education, lower secondary education, higher secondary education, and higher vocational college/university). Household income was divided in ten levels (<500 euro; between 500 and 2100 by steps of 200 Euro increase; >2100 euro) and was consecutively adjusted for the number of persons in the household.

Acculturation (the added explanatory factor) was operationalized into 5 domains: (1) mastery of Dutch language as a proxy for contact with native Dutch according to the model of Berry, (2) religiosity, (3) attitudes on care for family, (4) attitudes on male-female roles, and (5) attitudes on family values according to Ester. Dutch language proficiency was evaluated among Turkish and Moroccan elderly by three questions: (1) when someone talks to you in Dutch, are you able to understand (yes often, yes sometimes, no); (2) do you have difficulty in speaking Dutch (yes often, yes sometimes, no); (3) when you read a Dutch paper or a letter do you have difficulty in understanding (yes often, yes sometimes, no). A summated score was calculated which was subsequently recoded in 3 categories indicating mastery of Dutch language (1) poor, (2) mediocre, (3) good. Dutch language proficiency was not measured directly among Dutch, nor among Surinam and Antillean elderly who fluently speak Dutch because of their colonial background. As a proxy we asked whether the last time you went to the GP you were able to understand fully the GP (yes/no). If no, proficiency was considered mediocre; if yes, good.

Religiosity was measured by asking whether one considers oneself as belonging to a religion, and if yes, how frequently one attends religious meetings (every day, at least once a week, at least once a month, once or several times a year, almost never). Attitudes regarding care for family, male/female roles and family values were assessed by means of 14 propositions, e.g., children should take care of their parents when they are old, an education is more important for boys than for girls (agree, partly agree/partly disagree, do not agree). A summated score was calculated which was subsequently recoded in 3 categories indicating (1) traditional, (2) moderate traditional, or (3) modern attitudes on family care, male/female roles and family values.

### Analysis

First we described the respondent groups by socio-demographic and socio-economic status, acculturation, and self-perceived health according to ethnic background. Next we compared health care utilisation according to ethnic background, for each type of health service separately. The impact of determinants on specific utilisation (yes/no) was evaluated with logistic regression. First, we determined crude odds ratios (ORs) per ethnic group for the use of the five separate health care services. Second, we added the intended set of explanatory variables which might explain ethnic background effects to the extent present (self-perceived health, age, sex and socio-economic status, acculturation). This two-step procedure should reveal whether important 'unexplainable' ethnic differences remain. The analyses were performed using SPSS 13.0 for Windows. A two-sided test approach was chosen, where a p-value of 0.05 was considered a significant difference.

## Results

Immigrant elderly have a lower socio-economic position as indicated by their low educational and income level. Particularly Turkish and Moroccan elderly have a lower educational and income level compared to the native Dutch as well as Surinamese and Antilleans (see Table 1). As expected, there are also large differences in Dutch language proficiency: Surinamese and Antilleans are overall Dutch speaking while language proficiency among Turks and Moroccans on average is mediocre to poor. Compared to the native Dutch, immigrant elderly report more religious participation, particularly the Turkish and Moroccan group, and more often have a traditional attitude on family care, male/female roles and family values. Turkish, Moroccan and Surinamese elderly more often report a poor self-assessed health and more chronic conditions. Moreover, Turkish and Moroccan elderly more often report poor mental health.

**Table 1:** Socio-demographic and socio-economic status, acculturation and self-perceived health by ethnic background in the Netherlands (2003).

	***NETH*** ***(n = 304)***	***TURK*** ***(n = 307)***	***MOROC*** ***(n = 284)***	***SURI*** ***(n = 308)***	***ANTIL*** ***(n = 300)***	*p-value
**Socio-demographics**						
Age: 55-64 y (%)						0.904
Men	47.1	51.3	43.8	45.0	48.6	
Women	47.3	49.3	51.8	50.5	51.9	
**Socio-economic status**						
No education (%)	17.3	70.5	94.0	37.2	39.0	<0.001
Primary education (%)	14.0	12.5	3.2	11.9	9.9	
Lower secondary education (%)	33.0	14.9	0.7	21.8	19.9	
Higher secondary education (%)	20.3	1.0	1.8	17.2	17.0	
Higher vocational college/university (%)	15.3	1.0	0.4	11.9	14.2	
Standardised income (€/mnth, mean (sd))	1226 (497)	708 (215)	571 (193)	952 (425)	967 (500)	<0.001
**Acculturation**						
Mastery of Dutch language (%)						<0.001
Poor	0.0	48.2	44.7	0.0	0.0	
Mediocre	1.3	48.5	49.6	2.4	1.6	
Good	98.7	3.3	5.6	97.6	98.4	
Religious (%)	47.2	97.7	99.6	90.5	88.3	<0.001
Attendance religious meetings (%)						<0.001
Every day	0.7	26.5	27.5	2.0	2.7	
At least once a week	14.5	23.8	39.8	29.7	26.3	
At least once a month	6.9	12.9	4.2	16.7	15.7	
Once or several times a year	10.6	15.6	6.3	21.9	22.3	
Almost never	14.5	18.9	21.8	20.3	21.3	
Attitudes on care for family (%)						<0.001
Traditional	3.6	40.7	55.9	12.1	21.5	
Moderate traditional	36.3	48.3	41.6	57.0	47.0	
Modern	60.1	10.9	2.5	30.9	31.5	
Attitudes on male-female roles (%)						<0.001
Traditional	13.9	47.4	45.4	13.7	8.1	
Moderate traditional	29.7	35.1	25.7	35.9	36.2	
Modern	56.4	17.5	28.9	50.3	55.7	
Attitudes on family values (%)						
Traditional	11.3	30.7	36.2	20.9	14.3	<0.001
Moderate traditional	61.6	58.1	63.1	65.9	70.4	
Modern	27.2	11.2	0.7	13.2	15.3	
**Health status**						
No. of self-rated chronic conditions (%)						<0.001
0	28.0	6.2	10.9	17.5	24.3	
1-2	42.8	29.6	34.5	41.9	51.7	
≥ 3	29.3	64.2	54.6	40.6	24.0	
MCS SF-12, mean (sd)	51.7 (11.4)	41.6 (11.6)	42.0 (10.0)	46.2 (12.9)	49.7 (11.1)	<0.001
Self-rated health (%)						<0.001
Excellent	2.0	0.0	0.0	0.8	6.2	
Very good	4.0	1.2	0.0	0.8	1.0	
Good	38.6	17.9	8.6	21.7	27.8	
Fair	43.6	48.8	60.4	48.8	59.8	
Poor	11.9	32.1	30.9	27.9	5.2	

* χ^2 ^test was performed

The prevalence of immigrants consulting a GP is significantly higher than among Dutch elderly (see Table 2). Use of hospital care is about equal among groups. Turkish and especially Moroccan elderly use physical therapy and homecare services significantly less frequent.

**Table 2:** Self-reported health care use according to ethnic background in the Netherlands (2003).

**Health care use (%)**	**NETH (N = 304)**	**TURK (N = 307)**	**MOROC (N = 284)**	**SURI (N = 308)**	**ANTIL (N = 300)**	*p-value
GP	48.3	72.2	70.1	68.9	60.3	<0.001
Outpatient specialist	37.2	34.9	38.0	44.8	36.2	0.102
Hospital admission	14.5	19.8	13.2	18.8	14.5	0.106
Physical therapy	26.7	22.7	17.3	29.6	26.8	0.006
Homecare	21.7	11.7	3.6	24.2	14.3	<0.001

* χ^2 ^test was performed

Table 3 shows that more self-rated chronic conditions (OR 1.44; CI 1.28-1.62), worse self-rated health (OR 1.51; CI 1.27-1.80) and modern attitudes on male-female values (OR 1.12; CI 0.91-1.37) all contribute significantly to GP services use. In other words: for each extra chronic condition [OR1.44], the probability of GP services use rises 44%. Outpatient specialist services too are explained by more self-rated chronic conditions (OR 1.49; CI 1.34-1.67), worse self-rated health (OR 1.57; CI 1.31-1.88), and additionally by higher age (OR 1.02; CI 1.00-1.04) and good Dutch language proficiency (OR 1.46; CI 1.18-1.81). The same variables, with gender, significantly explain the presence of at least one hospital admission last year self-rated chronic conditions (OR 1.53 CI 1.33-1.76), self-rated health (OR 1.53; CI 1.20-1.94), gender (OR 1.66 CI 1.16-2.36) and good Dutch language proficiency (OR 1.33 CI 1.03-1.73). Physical therapy utilisation depends on the number of self-rated chronic conditions (OR 1.35; CI 1.20-1.53), worse self-rated health (OR 1.43; CI 1.17-1.74), male gender (OR 0.60; CI 0.45-0.81), higher age (OR 0.98; CI 0.96-1.00) and good Dutch language proficiency (OR 1.71; CI 1.35-2.18). Finally, home care utilisation is explained by the number of self-rated chronic conditions (OR 1.32; CI 1.13-1.55), self-rated health (OR 1.77; CI 1.36-2.31), gender (OR 0.53; CI 0.36-0.78), age (OR 1.09; CI 1.07-1.12) and good Dutch language proficiency (OR 2.49; CI 1.80-3.46). The remaining role of ethnic group after the above adjustments for health, socio-economic and socio-cultural background is: a significantly low OR regarding outpatient specialist use for Turkish and Moroccan elderly (OR 0.21; CI 0.09-0.46 and OR 0.31; CI 0.14-0.67), for Moroccan elderly regarding hospital admission (OR 0.28; CI 0.10-0.78) and again for Turkish and Moroccan elderly regarding home care (OR 0.19; CI 0.05-0.68/OR 0.07; CI 0.02-0.29). No substantial interaction effects between ethnic background and need factors were found.

**Table 3:** Self-reported use of GP care, outpatient care, hospital care, physical therapy, and home care, explained by ethnic background.

	**GP**	**Outpat. specialist**	**Hospital admiss**.	**Physical therapy**	**Homecare**
N	1090	1088	1084	1085	1076
***Crude analysis***					
Ethnic background (Dutch = ref)					
-Turkish	2.78(1.98-3.90)***	0.91(0.65-1.26)	1.45(0.95-2.23)	0.80(0.55-1.16)	0.48(0.31-0.75)***
-Moroccan	2.50(1.78-3.51)***	1.04(0.74-1.45)	0.89(0.56-1.43)	0.57(0.39-0.86)**	0.13(0.07-0.27)***
-Antillean	1.63(1.17-2.25)***	0.96(0.69-1.34)	1.00(0.63-1.57)	1.01(0.70-1.44)	0.61(0.40-0.93)*
-Surinamese	2.36(1.70-2.29)***	1.37(0.99-1.90)	1.37(0.89-2.10)	1.16(0.81-1.64)	1.15(0.79-1.69)
					
***Adjusted analysis***					
Self-reported number of chronic conditions in respondent (cf. prespecified list; range 0 - 11)	1.44 (1.28-1.62)***	1.49 (1.34-1.67)***	1.53 (1.33-1.76)***	1.35 (1.20-1.53)***	1.32 (1.13-1.55)***
Self-rated general health (range: 1 to 5; 1 = excellent)	1.51 (1.27-1.80)***	1.57 (1.31-1.88)***	1.53 (1.20-1.94)***	1.43 (1.17-1.74)***	1.77 (1.36-2.31)***
Self-rated mental health (range: 0 to 100; the higher the score the better the mental health)	1.00 (0.99-1.01)	0.99 (0.98-1.01)	0.99 (0.98-1.01)	1.01 (1.00-1.02)	1.00 (0.98-1.01)
Gender (male = 1, female = 2)	0.83 (0.63-1.11)	0.91 (0.70-1.20)	1.66 (1.16-2.36)**	0.60 (0.45-0.81)*	0.53 (0.36-0.78)***
Age (continuous in years)	1.00 (0.98-1.02)	1.02 (1.00-1.04)*	1.02 (0.99-1.04)	0.98 (0.96-1.00)***	1.09 (1.07-1.12)***
Educational level (no/primary education vs secondary and higher education)	1.17 (0.83-1.67)	1.16 (0.83-1.63)	0.96 (0.62-1.48)	1.03 0.71-1.49)	1.41 (0.90-2.21)
Standardized income (continuous in Euros)	1.00 (0.99-1.00)	1.00 (0.99-1.00)	1.00 (0.99-1.00)	1.00 (1.00-1.01)	1.00 (1.00-1.01)
Good Dutch language proficiency	1.10 (0.88-1.39)	1.46 (1.18-1.81)***	1.33 (1.03-1.73)*	1.71 (1.35-2.18)***	2.49 (1.80-3.46)***
Modern attitudes on care for family	0.98 (078-1.23)	1.01 (0.81-1.25)	1.06 (0.81-1.40)	0.90 (0.71-1.14)	1.22 (0.91-1.65)
Modern attitudes on male-female roles	1.12 (0.91-1.37)*	1.04 (0.86-1.30)	0.86 (0.67-1.11)	0.86 (0.69-1.07)	0.82 (0.62-1.09)
Modern attitudes on family values	0.74 (0.58-0.96)	1.04 (0.82-1.33)	1.23 (0.91-1.66)	0.93 (0.72-1.22)	1.20 (0.86-1.67)
Religiosity	1.06 (0.97-1.16)	0.99 (0.90-1.08)	0.93 (0.83-1.04)	0.91 (0.83-1.01)	1.07 (0.95-1.21)
Ethnic background (Dutch = ref)					
- Turkish	0.54 (0.25-1.18)	0.21 (0.09-0.46)***	0.40 (0.15-1.08)	0.67 (0.29-1.55)	0.19 (0.05-0.68)**
- Moroccan	0.57 (0.26-1.24)	0.31 (0.14-0.67)**	0.28 (0.10-0.78)*	0.50 (0.21-1.18)	0.07 (0.02-0.29)***
- Antillean	1.06 (0.68-1.66)	0.84 (0.53-1.34)	1.07 (0.58-1.96)	0.78 (0.47-1.24)	0.67 (0.37-1.24)
- Surinamese	1.34 (0.85-2.13)	0.67 (0.40-1.02)	0.92 (0.51-1.65)	0.88 (0.55-1.42)	0.90 (0.52-1.58)

*p < 0.05, **p < 0.01, ***p < 0.001

Comparison of crude analysis (ethnic background only as independent) and full analysis (a theoretically defined set of explanatory factors is added to the ethnic background variable). Odds Ratios and 95% Confidence Intervals obtained with univariate (crude) and multivariate (adjusted) logistic regression analysis.

## Discussion

This study among the four largest elderly immigrant groups in the Netherlands shows that substantial ethnic disparities exist in self-rated chronic conditions, self-rated mental health and self-rated overall health, with Turkish elderly being the worst off. Even more remarkable are the ethnic disparities in health care utilisation: use of GP services is higher among all immigrant groups, while use of physical therapy and home care is low to absent. Antilleans show a pattern in between the remaining three immigrant groups and the indigenous group. Health status (need factor) shows high explanatory power for all types of utilisation across all ethnic groups; however, income and educational level, both enabling factors, provide no additional explanation. These factors apparently are indirectly related to different utilisation patterns *through *their effect on health.

Acculturation, the concept we introduced as an additional predisposing factor in this context, appeared partly relevant. The instrumental role of language proficiency was remarkable: the ability of immigrant elderly to speak good Dutch has large impact on ethnic differences in secondary and tertiary health care use; e.g. the use of home care, which is typical for chronic conditions, increases with 150% if proficient. No other aspects of acculturation beyond language proficiency played a prominent role.

Without additional medical information it is impossible to set a threshold criterion to define over- or underutilisation of care; our analysis compares lower or higher utilisation compared to the reference use of the indigenous group. This interpretational uncertainty is particularly important in case of GP use by Turkish and Moroccan elderly, where the large overutilisation of GP care changes into strong underutilisation after taking our explanatory factors into account (fivefold reduction due to adjustment). Despite this uncertainty we believe that our data are suggesting underutilisation of all care except GP care.

Our study reveals inequalities among elderly immigrants. A recent study by Poort et al. [32] investigated the health care use of Turkish and Moroccan elderly (55-74 y) in Amsterdam. Their results can be compared validly with ours, showing similar patterns of these two immigrant groups. Acculturation and language competence was not part of that study. There are some limitations and therefore cautious interpretation is required. First, this study is based on self-reports of health status and of health care use. Regarding health care use, a study by Reijneveld showed that self-reports of hospitalisation and physical therapy provide fairly valid estimations in cross-cultural research [33]. Regarding health status, however, verified medical diagnosis information was lacking; this would be especially relevant to judge over- versus underutilisation in GP and specialist care.

Secondly, except for GP use, health care utilisation data did not provide quantitative information on the intensity of treatment, reflecting differential severity of medical conditions.

Thirdly, our ethnic coding could be challenged. We deliberately used recorded country of birth as indicator of immigrant background. As opposed to self-assessment this offers a double advantage: high reliability and lack of missing information. While it results in culturally homogenous groups for Moroccans and Turks, it covers relevant cultural differences in the Antillean and Surinamese group. The latter includes different groups such as Creole and Hindustani populations.

Fourthly, although language proficiency is a straightforward instrumental variable to explain a considerable amount of the disparities, the mechanisms behind it are unclear. Insufficient communication of need is a direct pathway, but language incompetence may also impair knowledge on health and health care services in the host country. We measured language proficiency among native Dutch, Surinamese and Antilleans with a proxy, namely whether they were able to understand their GP. We cannot exclude that with this proxy a broader concept of health literacy instead of only language proficiency was measured. The lack of explanatory power of the remaining acculturation factors does not exclude a role for specific factors: immigrants could prefer making use of informal care instead of home care, because they may consider these services not adapted to their needs, or because they expect care from their family. Here, supportive qualitative research should add to our quantitative results [34-36].

Finally, non-response rates may affect our results. The age/sex distributions in our samples are as expected due to the stratified sampling procedure, indicating no selective non-response in this regard. The most frequent reason for non-response is absence of the respondent at the address at the time of visit and to a lesser extent being ill and outright refusal. The reasons for non-response did not differ systematically according to ethnic background. Hence, while non-response could affect disease prevalence in the responding group (lower), it is unlikely that this will affect associations of determinants across groups, as the pattern of selection is similar across *all *groups. We are aware of two thorough studies on the effect of selective non-response. One study conducted by Statistics Netherlands, the organisation being responsible for national surveys, reported on to the presence of ethnic-related non-response in a key survey [37]. The approach rested on sophisticated weighting experiments, using personal administrative data. Statistics Netherlands reported grossly unaffected prevalences of intended key indicators (including subjective health). Moreover, their report showed that adjustment by weighting for ethnic-specific imbalance of determinants of those indicators, for which national numbers were known, yielded negligible effects on the aggregate indicator score distribution. Apparently the association between key variables and determinants is the same among non-respondents and respondents. The second study is the Amsterdam Born Children and their Development-study on ethnicity related perinatal health [38]. This study was able to pursue an empirical approach of non-response effects: data on non-respondents (outcomes and determinants) could be retrieved anonymously from national registries. Again it was observed that the prevalence of outcomes and determinants (like e.g. education) were affected due to selective participation. However, associations and results from regression analysis for a number of known perinatal relations of social and medical determinants with perinatal health were not affected to any relevant degree. Moreover, a study by Reijneveld et al [3] showed that specialist and paramedic care use is lower among non-respondents than among respondents, unexplained by demographic and socio-economic factors, including country of birth. This implies that our estimates of the use of outpatient care and of physical therapy use may be to low, but that differences between native and immigrant elderly probably are unaffected.

To conclude, our methodological choice to extend the standard Anderson model with acculturation paid off: in the context of analysis of ethnic disparities in health care utilisation it provided an explanatory tool limited to the introduction of language competence as important instrumental variable.

While the first part of our hypothesis (health disparities translate into utilisation disparities) could be confirmed, our hypothesis on the role of other determinants has to be revised. Rather than the conventional socio-economic and educational factors, language proficiency was the single instrumental predisposing (but probably also enabling) factor.

The resulting pattern of systematic and sizable underutilisation is a challenge for health care providers and policy makers. Non-Dutch speaking patients should definitively be recognized as a high-risk group. Generally, intervention targets are present at both sides: new comers should be offered facilities to learn and improve language skills, while first generation elderly immigrants primarily rely on peer educators.

## Competing interests

The authors declare that they have no competing interests.

## Authors' contributions

SD and MF participated in the design of the study; SD performed the statistical analysis with GK and SD drafted the manuscript. EB and MF commented on the draft. GB assisted in drafting the manuscript. All authors read and approved the final manuscript.
